# The 21st Biennial congress of the South African Society of Nuclear Medicine conference report

**DOI:** 10.4102/jcmsa.v4i1.377

**Published:** 2026-07-28

**Authors:** Bawinile P. Hadebe, Mariza Vorster

**Affiliations:** 1 Department of Nuclear Medicine, School of Medicine, University of KwaZulu-Natal, Durban, South Africa; 2 Inkosi Albert Luthuli Central Hospital, Durban, South Africa

**Sponsor disclaimer:** We would like to express our heartfelt gratitude to Prof. Mike Satheke, Head of the Nuclear Medicine department and President and CEO of Nuclear Medicine Research Infrastructure (NuMeRI), for the generous funding that made publishing this conference report possible.

## Introduction

The 21st Biennial Congress of the South African Society of Nuclear Medicine (SASNM) was convened from Thursday, 31 July 2025 to Saturday, 02 August 2025 at the Premier Hotel uMhlanga in Durban, South Africa. The congress was held under the overarching theme ‘Patient-Centred Precision Care with Pride’, reflecting the Society’s ongoing commitment to advancing nuclear medicine practices that are both clinically effective and patient focused.

The congress commenced with two parallel tracks on 31 July 2025. Track One featured a hands-on practical workshop specifically designed for radiographers and radiopharmacists. The session focused on aseptic radiolabelling techniques, quality control procedures and the administration of contrast agents in positron emission tomography/computed tomography (PET/CT) imaging. This initiative aimed to enhance technical competencies and promote best practices in clinical radiopharmacy. Track Two featured an examination preparation workshop led by Prof. John Buscombe, primarily designed for nuclear medicine registrars and open to nuclear physicians. The session offered valuable updates on theranostic applications in bone disease, liver disease, neuroendocrine tumours and prostate cancer.

The scientific programme was structured around four key thematic areas: nuclear oncology and theranostics, brain imaging, ethics, regulation and radiopharmacy, as well cardiac and paediatric imaging. Notably, the oncology sessions highlighted recent advances in theranostic approaches and the need to further develop these strategies, especially in relation to cancers that predominantly affect female patients. In addition, a panel discussion featuring a representative from the South African Health Products Regulatory Authority (SAHPRA) addressed the regulatory requirements for establishing and operating a nuclear medicine facility.

Prof. John Buscombe received a lifetime achievement award in recognition of his longstanding dedication to registrar training and his impactful contributions to nuclear medicine in South Africa. Another new feature introduced at this congress was a wellness-focused fun run along the promenade, aimed at promoting physical health and reinforcing the importance of maintaining a balanced work–life dynamic.

A total of 39 abstracts were accepted for presentation following peer review. These comprised 14 oral presentations and 25 poster presentations. The diversity and quality of submissions reflected the robust research activity within the South African nuclear medicine community. The following individuals were awarded best abstracts in recognition of their outstanding contribution towards the field of nuclear medicine in the country: Dr Bianca Berndorfler, Ms Kaluzi Banda, Ms Yonwaba Mzizi, Ms Motshidisi Ashleigh Mokoena for Oral presentations, and Dr Thembelihle Nxasana, Ms Pryaska Goorhoo, Mr Branden Tshijolo and Mr Jayed Oliver for poster presentations.

