# Supplementary material for: The 21st Biennial congress of the South African Society of Nuclear Medicine conference report
Source: J Coll Med S Afr. 2026 Jul 28;4(1):377. doi: 10.4102/jcmsa.v4i1.377 (PMC13443484; doi:10.4102/jcmsa.v4i1.377)
Supplement: Related texts

# Abstract: Distinct metabolic patterns of neuropsychiatric systemic lupus erythematosus on hierarchical cluster analysis

**Published:** 2026-07-28

**Authors:** B.D. Berndorfler, J.M. Warwick, P. Dupont, R. du Toit, A. Engelbrecht, T. Jardine, P. Sadhai, T. Sabela, V. Anopuechi-Clarkson, A.G.G. Doruyter

**Affiliations:** 1 Division of Nuclear Medicine, Faculty of Medicine and Health Sciences, Stellenbosch University, Cape Town, South Africa; 2 KU Leuven Brain Institute, Leuven, Belgium; 3 Nuclear Medicine Research Infrastructure (NuMeRI) Node for Infection Imaging, Stellenbosch University, Cape Town, South Africa

## Abstract

**Background:**

Neuropsychiatric systemic lupus erythematosus (NPSLE) is a complex autoimmune disorder with diverse neurological manifestations. Diagnosis is challenging because of overlapping symptoms and a lack of specific biomarkers. Functional neuroimaging, particularly fluorodeoxyglucose positron emission tomography/computed tomography (FDG PET/CT), offers a promising avenue for identifying cerebral metabolic patterns that may correlate with disease activity and clinical subtypes.

**Methods:**

Forty-nine patients meeting SLEDAI-2K criteria and presenting with neuropsychiatric symptoms underwent FDG PET/CT imaging. Brain metabolic activity was quantified using the Brainnetome atlas. Hierarchical cluster analysis was applied to identify distinct metabolic profiles. Clinical parameters including ESR, haemoglobin and autoantibody levels were compared across clusters.

**Results:**

Two distinct metabolic clusters were identified. Cluster 2 exhibited hypermetabolism in the basal ganglia, thalami, medial temporal lobes and cerebellum, alongside cortical hypometabolism. Patients in this cluster had significantly higher SLEDAI-2K scores, more frequent psychosis, stroke and vasculitis, and elevated anti-ribosomal P and anti-cardiolipin antibodies. The metabolic signature resembled autoimmune encephalitis, suggesting a shared pathophysiological mechanism. Cluster 1 showed less pronounced metabolic changes and milder clinical features.

**Conclusion:**

Fluorodeoxyglucose positron emission tomography/computed tomography combined with hierarchical cluster analysis reveals distinct cerebral metabolic patterns in NPSLE, offering a novel approach to disease classification. Cluster 2 may represent a more aggressive subtype requiring intensive immunosuppressive therapy. These findings support the integration of functional imaging into routine NPSLE evaluation and highlight the potential of imaging biomarkers in guiding personalised treatment strategies.

# Abstract: Hope through radioactive iodine: Treating multifocal encapsulated follicular variant of papillary thyroid carcinoma with bone metastases at Chris Hani Baragwanath Academic Hospital

**Published:** 2026-07-28

**Authors:** M.N.B. Cossie, K.J. Kobela, A. Ismail

**Affiliations:** 1 Department of Nuclear Medicine and Molecular Imaging, University of the Witwatersrand, Johannesburg, South Africa

## Abstract

**Background:**

Encapsulated follicular variant of papillary thyroid carcinoma (EFVPTC) is a subtype of papillary thyroid carcinoma (PTC) marked by follicular architecture and reticular activating system (RAS) mutations. While non-invasive forms may be reclassified as non-invasive follicular thyroid neoplasm with papillary-like nuclear features (NIFTP), invasive and multifocal variants pose higher risks of recurrence and metastasis. Bone metastases, although uncommon, complicate treatment because of poor radioactive iodine (RAI) uptake and prognosis. To evaluate the efficacy of RAI therapy in treating multifocal invasive EFVPTC with bone metastases and to highlight the importance of individualised treatment and monitoring in complex metastatic thyroid cancer cases.

**Methods:**

A 61-year-old hypertensive male presented with a large left neck mass confirmed via fine needle aspiration (FNA) as a malignant follicular variant of PTC. Computed tomography imaging revealed a thyroid mass with tracheal displacement and a T3 vertebral lytic lesion. After total thyroidectomy, multifocal invasive EFVPTC was diagnosed, with a largest lesion of 93 mm, angioinvasion and lymphovascular invasion (T3a, Nx, stage II). Despite surgery, high thyroglobulin levels and inadequate thyroid stimulating hormone (TSH) rise prompted referral for nuclear medicine evaluation. Tc-99m Pertechnetate imaging showed no residual thyroid tissue, but single photon emission computed tomography (SPECT)/CT identified lytic bone metastases at T12 and sclerotic lesions at L2 and L3.

**Results:**

Initial I-131 diagnostic scans revealed widespread metastases. A cumulative dose of 500 mCi I-131 was administered in 2024. After defaulting Eltroxin, the patient returned with bone pain. A 5-month post-therapy scan showed reduced disease burden, prompting a second dose of 300 mCi I-131 (total 700 mCi). Follow-up scans indicated partial response and no new lesions. Thyroglobulin levels showed a downward trend, and the patient was placed on suppressive Eltroxin therapy.

**Conclusion:**

This case underscores the utility of RAI therapy in managing metastatic EFVPTC, particularly with bone involvement. Despite initial challenges, tailored nuclear medicine intervention led to measurable improvement. Continuous imaging and biochemical monitoring remain vital for guiding treatment and achieving favourable outcomes in complex thyroid cancer cases.

# Abstract: Human papillomavirus-associated vulvar cancer: Impact of HIV status on fluorodeoxyglucose positron emission tomography/computed tomography

**Published:** 2026-07-28

**Authors:** E.A. Onyango, J.L. Butt, J. Warwick, B. Berndorfler

**Affiliations:** 1 Division of Nuclear Medicine, Department of Medical Imaging and Clinical Oncology, Stellenbosch University, Cape Town, South Africa; 2 Department of Obstetrics and Gynaecology, Stellenbosch University, Cape Town, South Africa

## Abstract

**Background:**

Fluorodeoxyglucose positron emission tomography/computed tomography (FDG PET/CT) has been utilised in the management of vulvar malignancies; however, not much is known about using FDG PET/CT in vulvar cancers associated with human papillomavirus (HPV), especially in the context of human immunodeficiency virus (HIV) comorbidity. This study aims to describe the FDG PET/CT patterns in patients diagnosed with HPV-dependent vulvar cancer, evaluating any association between HIV status across age groups, Fédération Internationale de Gynécologie et d’Obstétrique (FIGO) stage, primary treatment and indication for FDG PET/CT scan.

**Methods:**

Data were collected between 2012 and 2023 among women 18 years and above diagnosed with vulvar cancer undergoing FDG PET/CT scan at Tygerberg hospital. A total of 73 participants were enrolled with HPV-dependent disease (45 HIV positive and 28 HIV negative). Chi-square and Fishers’ exact tests were used to assess statistical associations.

**Results:**

HIV-positive patients were significantly younger compared to the HIV-negative group (mean age 38.0 ± 6.9 vs 54.0 ± 13.5, *p* < 0.0001). FIGO stage (*p* = 0.547), primary treatment (*p* = 0.370) and reason for PET referral (*p* = 0.644) revealed no significant difference between the HIV-positive and HIV-negative individuals. The proportion of patients with positive lymph nodes on PET did not differ between HIV-positive (97.8%) and HIV-negative (89.3%) individuals (*p* = 0.121). Similarly, proportions of individuals with positive lymph nodes in inguinal, pelvic, common iliac and extra-pelvic stations showed were not significantly different (*p* = 0.681). The proportion of patients with distant metastases also did not differ between HIV-positive and HIV-negative individuals (2/45 vs 2/28, *p* = 0.195).

**Conclusion:**

HIV-positive patients with HPV-associated vulvar cancer were younger than HIV-negative patients but showed no difference in nodal status or detection of distant metastases on FDG PET/CT.

# Abstract: Triple-negative breast cancer and F-18 fluorodeoxyglucose positron emission tomography/computed tomography

**Published:** 2026-07-28

**Authors:** A. Venter, K. Purbhoo, M.D.T. Vangu

**Affiliations:** 1 Nuclear Medicine Department, University of the Witwatersrand, Charlotte Maxeke Johannesburg Academic Hospital, Johannesburg, South Africa

## Abstract

**Background:**

Triple-negative breast cancer (TNBC) is a biologically aggressive subtype of breast cancer lacking ER, PR and HER2 expression. Representing 15%–20% of breast cancer cases, TNBC is associated with early visceral metastasis and poor prognosis. Its metastatic spread often targets the lungs, liver and brain. F-18 fluorodeoxyglucose positron emission tomography/computed tomography (FDG PET/CT) is a valuable imaging modality for assessing tumour metabolism and disease extent, playing a pivotal role in TNBC management.

**Aims:**

This study aimed to evaluate the metastatic distribution and metabolic activity of TNBC using F-18 FDG PET/CT in patients referred for staging, restaging or therapy response assessment.

**Methods:**

A retrospective review was conducted on 31 patients with histologically confirmed TNBC who underwent F-18 FDG PET/CT between January 2016 and September 2022. Data collected included patient demographics, immunohistochemical markers (ER, PR, HER2, Ki-67%), disease spread patterns and standardised uptake value maximum (SUVmax) from PET/CT imaging.

**Results:**

Among the 31 TNBC patients, PET/CT was primarily used for restaging (68%), followed by therapy response evaluation (29%) and initial staging (3%). Disease was detected in 81% of cases. Although not statistically significant, lung metastases were the most common. Triple-negative breast cancer demonstrated high proliferative activity, with a mean Ki-67 index of 70% (range: 40% – 80%) and elevated SUVmax values averaging 13.5 (range: 7.2–19). Ki-67 was significantly associated with TNBC (*p* = 0.0001), while SUVmax showed borderline significance (*p* = 0.0564).

**Conclusion:**

Triple-negative breast cancer’s aggressive biology is reflected in its high metabolic activity and elevated Ki-67 levels. F-18 FDG PET/CT effectively identifies metastatic disease, particularly in the lungs, and supports clinical decision-making in staging and treatment monitoring. Future prospective studies and novel PET tracers may enhance diagnostic precision and therapeutic planning for TNBC.

# Abstract: Intra-individual comparison of [^68^Ga]Ga-PSMA-11 PET/CT and [^99m^Tc]Tc-PSMA-GCK01/RHN001DX SPECT/CT to identify a suitable imaging surrogate for phenotyping prior to PSMA-RLT

**Published:** 2026-07-28

**Authors:** H. Ndlovu, E. Mamlins, A. Feisthaue, J. Cardinale, C. Kratochwil, J. Kabunda, S. Sibiya, Y. Mzizi, A. Mdlophane, S. Mdanda, M. Shungube, C. Kaoma, K. Ramonaheng, M. Qebetu, C. Davis, K. Mokoala, F.L. Giesel, M. Sathekge

**Affiliations:** 1 Nuclear Medicine Research infrastructure (NuMeRI), Department of Nuclear Medicine, University of Pretoria and Steve Biko Academic Hospital, Pretoria, South Africa; 2 Department of Nuclear Medicine, Medical Faculty and University Hospital Düsseldorf, Heinrich-Heine-University Düsseldorf, Germany; 3 Department of Nuclear Medicine, University Hospital Heidelberg, Heidelberg, Germany

## Abstract

**Purpose/Background:**

^177^Lu-PSMA-617 (Pluvicto^®^) is an established radioligand therapy (PSMA-RLT) for metastatic castration-resistant prostate cancer (mCRPC), offering improved survival over standard treatments. The Phase III VISION trial required PSMA-positive lesions with a lesion-to-liver ratio > 1 on ^68^Ga-PSMA-11 positron emission tomography (PET) imaging for therapy eligibility. In regions lacking PET access, single photon emission computed tomography (SPECT)/CT imaging with ^99m^Tc-labelled PSMA ligands may serve as an alternative. This study aimed to evaluate whether ^99m^Tc-PSMA-GCK01 can provide comparable imaging surrogates for PSMA phenotyping relative to ^68^Ga-PSMA-11.

**Methods:**

Six patients were prospectively imaged using both ^99m^Tc-PSMA-GCK01 SPECT/CT and ^68^Ga-PSMA-11 positron emission tomography/computed tomography (PET/CT), with a minimum 48-h interval between scans. ^68^Ga-PSMA-11 was sourced from routine clinical production, while 99mTc-PSMA-GCK01 was synthesised with > 95% radiochemical yield and purity. Tracer uptake in malignant lesions and normal organs was assessed semi-quantitatively using spherical volumes of interest (VOIs). Three lesions per metastatic site (skeletal, lymphatic, visceral) were analysed. Tumour-to-background and tumour-to-blood pool ratios were calculated using mediastinal blood pool and pelvic fat references. SUVmax values from PET and counts from SPECT were normalised for direct comparison.

**Results:**

All imaging procedures were completed successfully with no adverse effects. Biodistribution patterns were similar across most organs. Kidney uptake was higher with 68Ga-PSMA-11 (median ratio 13.2) than with ^99m^Tc-PSMA-GCK01 (9.45), while liver uptake was slightly higher with ^99m^Tc-PSMA-GCK01 (median target-to-blood pool ratio 4.6 vs. 4.0). Lesion-to-liver ratios were lower with ^99m^Tc-PSMA-GCK01 (median 2.1, range 0.12–5.8) compared to ^68^Ga-PSMA-11 (median 4.9, range 1.3–8.3).

**Conclusion:**

^99m^Tc-PSMA-GCK01 SPECT/CT shows promise as a viable imaging surrogate for PSMA phenotyping prior to PSMA-RLT, especially in PET-limited settings. Liver uptake comparability supports its potential role in therapy eligibility assessment.

# Abstract: Evaluation of an automated synthesis method for [^18^F]fluoromisonidazole using an automated synthesis module in a South African hospital radiopharmacy using off-site produced fluoride-18

**Published:** 2026-07-28

**Authors:** J. Oliver, J. le Roux, S. Rubow

**Affiliations:** 1 Nuclear Medicine Division, Stellenbosch University, Tygerberg, South Africa; 2 Node for Infection Imaging, Stellenbosch University, Tygerberg, South Africa

## Abstract

**Background:**

[^18^F]FMISO is a positron emission tomography (PET) radiopharmaceutical used for hypoxia imaging and is the only agent of its kind with a dedicated monograph in the European Pharmacopoeia. Its clinical utility is well established, but local production remains limited. This study explored the feasibility of synthesising [^18^F]FMISO using commercially sourced [^18^F]fluoride in a cassette-based automated radiosynthesis module within a South African hospital radiopharmacy.

**Methods:**

Automated synthesis was performed using cassettes, reagent kits and precursor vials from approved suppliers. [^18^F]fluoride was procured from a commercial PET radionuclide provider. The synthesis followed a validated protocol involving trapping [^18^F]fluoride on a QMA cartridge, elution with tetrabutylammonium hydrogen carbonate and drying in acetonitrile at 95°C. The precursor reacted with the dried [^18^F]fluoride at 110°C for 10 min. The crude product was purified using SPE cartridges, filtered and buffered to the correct pH. Quality control adhered to Ph. Eur. Monograph 2459, and shelf-life was monitored over 8 h.

**Results:**

Five synthesis batches were completed. [^18^F]FMISO was successfully produced with high radiochemical purity (99.4 ± 0.5%). However, transport delays in receiving [^18^F]fluoride led to inconsistent starting activities. The average decay-corrected radiochemical yield was 27.4 ± 3.6%, and the final product activity averaged 1.7 ± 0.5 GBq. These yields were lower than that reported in literature, necessitating frequent synthesis runs to meet clinical demand, increasing costs and resource requirements.

**Conclusion:**

While [^18^F]FMISO synthesis using commercial [^18^F]fluoride is feasible and yields high-purity product, logistical challenges and low radiochemical yields limit its practicality for routine clinical use. Small-scale production enhances access in decentralised settings but incurs higher operational costs, highlighting the need for improved infrastructure or cyclotron integration to optimise efficiency.

# Abstract: Exploration of F-18 fluorodeoxyglucose positron emission tomography/computed tomography radiomic analysis of the primary tumour in locally advanced stage cervix cancer to predict more advanced disease

**Published:** 2026-07-28

**Authors:** J. Meyer, H. Burger, A. Doruyter, M. Mix, J. Warwick, C. Burger

**Affiliations:** 1 Nuclear Medicine Division, Department of Medical Imaging and Clinical Oncology, Stellenbosch University, Cape Town, South Africa; 2 Nuclear Medicine Research Infrastructure (NuMeRI Node) for Infection Imaging, Central Analytical Facilities, Stellenbosch University, Cape Town, South Africa; 3 Division of Radiation Oncology, Department of Medical Imaging and Clinical Oncology, Stellenbosch University, Cape Town, South Africa; 4 Department of Nuclear Medicine, University of Freiburg, Freiburg, Germany

## Abstract

**Background:**

Cervical cancer is the leading cause of cancer-related death in South African women, with a mortality of 1 in 6 diagnosed patients. Radiomics extract large amounts of quantified voxel-based data from medical images. Radiomic features of Fluorine-18 fluorodeoxyglucose positron emission tomography/computed tomography (FDG PET/CT) may serve as prognostic biomarkers and thereby assist management. This study aimed to determine whether PET radiomic features are correlated with more advanced disease.

**Methods:**

A retrospective study included women with potentially curable, locally advanced squamous cell cervical cancer who underwent baseline FDG PET/CT for staging and radiotherapy planning at Tygerberg Hospital, South Africa between September 2017 and December 2018. Participants were categorised into two groups based on the presence or absence of nodal metastases. The biological tumour volume of the primary lesion was segmented using FDG PET images. Radiomic features of the primary tumour were extracted using Matlab Radiomics toolbox. An exploratory comparison of the two groups was performed with simple parametric tests.

**Results:**

Seventy-four participants (median age 43 years) were included, 58 with and 16 without nodal metastases. Participants with lymph node metastases had larger tumours (*p* < 0.01) and a higher degree of texture heterogeneity, including features describing changes in grey levels between voxels (*p* < 0.01), but intensity of uptake was not statistically significant. Human immunodeficiency virus (HIV) status did not have a significant impact on the radiomic features (HIV-positive cases: *n* = 35, 44%).

**Conclusion:**

Fluorodeoxyglucose positron emission tomography radiomic analysis of the primary tumours in locally advanced cervical cancer showed that more advanced disease was associated with radiomic features such as larger size and intratumoural heterogeneity, which may be indicative of more aggressive disease and a poorer prognosis.

# Abstract: In vitro stability of commercially available ^99m^Tc-DTPA kits up to 24 hours

**Published:** 2026-07-28

**Authors:** B.W. Mekonnen, J.L. Holness, J.e le Roux, J. Oliver, M.M. Klein, J.M. Warwick

**Affiliations:** 1 Nuclear Medicine Division, Department of Medical Imaging and Clinical Oncology, Stellenbosch University, Cape Town, South Africa; 2 Nuclear Medicine Research Infrastructure (NuMeRI Node) for Infection Imaging, Stellenbosch University, Cape Town, South Africa

## Abstract

**Background:**

Radionuclide-based methods such as slope-intercept and single-sample plasma clearance are widely used for glomerular filtration rate (GFR) measurement. However, their accuracy diminishes in patients with expanded third spaces (e.g. ascites, pleural effusion, oedema) or impaired renal function. In such cases, delayed blood sampling – up to 24 h – is recommended. These protocols were originally developed using ^51^Cr-EDTA, but ^99m^Tc-DTPA is increasingly preferred. This shift raises concerns about the radiochemical stability of ^99m^Tc-DTPA over extended periods, which this study aims to evaluate.

**Methods:**

Three commercial ^99m^Tc-DTPA kits (Products A, B and C) were tested. Each was prepared with an average activity of 411 ± 13.67 MBq in 5 ml of 0.9% saline. Samples were incubated at 37 °C to simulate physiological conditions. Radiochemical purity (RCP) was assessed using thin layer chromatography (TLC) at 0 h, 2 h, 4 h, 6 h, and 24 h -post-preparation. Each kit was tested in triplicate, with an in-house RCP threshold of ≥ 95%.

**Results:**

Product A maintained high RCP, with 98.8 ± 1.7% at 6 h and 94.7 ± 2.6% at 24 h. Product B showed significant degradation, dropping from 86.7 ± 5.1% at 6 h to 20.2 ± 2.7% at 24 h. Product C demonstrated intermediate stability, with 98.6 ± 1.1% at 6 h and 85.7 ± 2.3% at 24 h. These findings highlight substantial variability in kit performance over time.

**Conclusion:**

The stability of ^99m^Tc-DTPA kits varies considerably, with Product A showing the most reliable performance for delayed sampling. These results suggest that kit selection is critical when adapting GFR protocols for extended sampling intervals. Further *in vivo* studies are needed to validate these findings and guide clinical practice in nuclear medicine.

# Abstract: Evaluating ^18^F-fluorodeoxyglucose positron emission tomography/computed tomography in the diagnosis and management of complicated paediatric tuberculosis

**Published:** 2026-07-28

**Authors:** D. Smith-Tsibolane, C. Burger, C. le Roux, A. Doruyter, P. Goussard

**Affiliations:** 1 Division of Nuclear Medicine, Department of Medical Imaging and Clinical Oncology, Stellenbosch University, Cape Town, South Africa; 2 Division of Radiodiagnosis, Department of Medical Imaging and Clinical Oncology, Stellenbosch University, Cape Town, South Africa; 3 Nuclear Medicine Research Infrastructure (NuMeRI Node) for Infection Imaging, Central Analytical Facilities, Stellenbosch University, Cape Town, South Africa; 4 Department of Paediatrics and Child Health, Faculty of Medicine and Health Sciences, Stellenbosch University, Cape Town, South Africa

## Abstract

**Background:**

Children account for approximately 20% of tuberculosis (TB) cases in South Africa and are particularly vulnerable to severe disease forms. Early diagnosis is essential to reduce morbidity, mortality and transmission. However, paediatric TB diagnosis is challenging because of its nonspecific presentation, paucibacillary nature and difficulty in obtaining diagnostic samples. Imaging is vital when microbiological confirmation is lacking. While chest X-rays and CT scans are commonly used, they have limitations in detecting active disease and mediastinal lymphadenopathy. Fluorodeoxyglucose positron emission tomography/computed tomography offers functional imaging of active inflammation, aiding diagnosis, staging and biopsy guidance.

**Methods:**

This retrospective study reviewed imaging, bronchoscopy, and microbiology data from children aged 3 months to 16 years who underwent fluorodeoxyglucose positron emission tomography/computed tomography (FDG PET/CT) at Tygerberg Hospital between January 2013 and October 2024. Positron emission tomography/computed tomography scans were interpreted by a nuclear physician and compared to diagnostic CT or low-dose CT from PET/CT. Volumes of interest were drawn for target lesions. A paediatric pulmonologist assessed clinical outcomes and PET/CT’s impact on management.

**Results:**

Thirty-four children (median age 7 years; 16 males; 4 human immunodeficiency virus (HIV) positive) were included. Most (68%) had prior diagnostic CT. Positron emission tomography/computed tomography identified 152 intrathoracic nodal sites versus 102 on CT. Extrapulmonary disease was detected in 50% of patients via PET/CT, compared to 18% on CT. Mean SUVmax values were 3.6 for mediastinal nodes, 2.6 for pulmonary lesions and 2.0 for extrapulmonary sites. Positron emission tomography/computed tomography confirmed persistent disease in 50%, identified diagnostic biopsy sites in 26%, excluded TB in 12%, guided surgery in 6%, flagged non-responders in 6% and confirmed recurrence in 3%.

**Conclusion:**

Fluorodeoxyglucose positron emission tomography/computed tomography significantly influenced clinical management, outperforming CT in detecting intrathoracic and extrapulmonary TB. Its ability to confirm disease activity and guide interventions underscores its value in complex paediatric TB cases.

# Abstract: A database for radiopharmacy in anglophone Africa

**Published:** 2026-07-28

**Authors:** L. Mosima, A.E. Manicum, B. Summers

**Affiliations:** 1 Department of Pharmaceutical Sciences, Sefako Makgatho Health Sciences University, Ga-Rankuwa, South Africa; 2 Department of Chemistry, Tshwane University of Technology, Pretoria, South Africa

## Abstract

**Background:**

Libraries of available infrastructure for nuclear medicine and related services in Africa are essential tools to inform governments and donating organisations of countries that lack and those with insufficient nuclear medicine services for proper allocation of resources and prioritisation. The study aimed to investigate the availability of radiopharmacies in English-speaking countries and develop a radiopharmacy database for this region.

**Method:**

Data were collected through a semi-structured, self-administered online questionnaire. The questionnaire was shared with nuclear medicine physicians, radiopharmacists, medical physicists and nuclear medicine technologists through different online and in-person platforms.

**Results:**

Out of 24 English-speaking African countries only 13 countries have nuclear medicine services (Cameroon, Ethiopia, Ghana, Kenya, Mauritius, Namibia, Nigeria, South Africa (SA), Sudan, Tanzania, Uganda, Zambia and Zimbabwe). The study found South Africa to have the largest nuclear medicine infrastructure, with four radiopharmaceutical manufacturing facilities and a state-of-the-art nuclear medicine research infrastructure NuMeRI. Thirteen public sector institutional radiopharmacies were recorded in SA. Because of the reluctance of the private sector to participate, only 14 of the 92 private SA facilities responded. Cameroon, Ghana, Mauritius, Uganda, Zambia and Zimbabwe have one radiopharmacy per country serving their entire nation. Kenya, Nigeria, Tanzania and Ethiopia each have two radiopharmacies per country, while Sudan has four radiopharmacies.

**Conclusion:**

A comprehensive database that includes institutional and standalone radiopharmacies for preparing and compounding small-scale radiopharmaceuticals, central radiopharmacies and radiopharmaceutical manufacturing facilities from English-speaking African countries was developed. This information, hosted by an organisation such as the International Atomic Energy Agency (IAEA), should lead to improved decision-making by policymakers when allocating resources and help medical practitioners refer patients for further investigation or treatment in nuclear medicine, as doctors can tell which imaging services are offered by nuclear medicine centres.

# Abstract: Segmentation of hypoxia in locally advanced cervical cancer on F-18 fluoromisonidazole positron emission tomography/computed tomography and comparison with the biological tumour volume on F-18 fluorodeoxyglucose positron emission tomography/computed tomography using affinity multimodality viewer

**Published:** 2026-07-28

**Authors:** C. Burger, A. Doruyter, H. Burger, J. Warwick

**Affiliations:** 1 Nuclear Medicine Division, Department of Medical Imaging and Clinical Oncology, Stellenbosch University, Cape Town, South Africa; 2 Nuclear Medicine Research Infrastructure (NuMeRI Node) for Infection Imaging, Central Analytical Facilities, Stellenbosch University, Cape Town, South Africa; 3 Division of Radiation Oncology, Department of Medical Imaging and Clinical Oncology, Stellenbosch University, Cape Town, South Africa

## Abstract

**Background:**

Cervical cancer remains the leading cause of cancer-related mortality among women in South Africa. Locally advanced cases are typically treated with chemoradiotherapy and brachytherapy. Tumour hypoxia contributes to radio-resistance and poor outcomes. Radiomics is quantitative analysis of medical imaging, which offers a promising avenue to explore tumour microenvironment features. This study investigates the potential of Fluorine-18 fluoromisonidazole (FMISO) positron emission tomography/computed tomography (PET/CT) radiomic features to predict overall survival in patients undergoing curative chemoradiotherapy, with a focus on developing a reliable segmentation pipeline.

**Methods:**

Women over 18 with locally advanced squamous cell cervical carcinoma were recruited to undergo FMISO PET/CT within 3 weeks of a standard FDG PET/CT used for radiotherapy planning. Both scans were reconstructed to 2 mm isotropic voxels and analysed using Affinity Multimodality Viewer v4.0.2. Tumour volumes were delineated on FDG PET using a validated segmentation method from Freiburg University. Hypoxic regions on FMISO PET were identified using established thresholding techniques. Co-registration of FDG and FMISO scans was performed to align anatomical and functional data.

**Results:**

Twenty patients were evaluated. The Affinity co-registration tool effectively aligned FDG and FMISO scans. However, distinguishing tumour boundaries from adjacent structures was challenging, particularly on FMISO PET. To ensure consistency, FDG-based tumour segmentation and inverted bladder activity masks were used to constrain FMISO tumour volumes. Scattered counts from bladder activity remained a limitation although expanding the bladder volume of interest (VOI) may mitigate this issue.

**Conclusion:**

A segmentation pipeline for FMISO PET/CT in cervical cancer was successfully developed, enabling comparable tumour volume analysis across modalities. While promising, further refinements are needed to address bladder-related artefacts. This work supports the integration of FMISO radiomics into prognostic modelling and personalised treatment planning for cervical cancer.

# Abstract: The optimal thyroglobulin cut off value for detecting fluorodeoxyglucose-avid disease in thyroglobulin-elevated negative iodine scintigraphy syndrome. A retrospective analysis

**Published:** 2026-07-28

**Authors:** N.D. Ndlovu, B.P. Hadebe, L.M. Harry, L. Gabela, N. Madi, T.S. Nxasana, S. Masikane, V. Pillay, M. Patel, M. Vorster

**Affiliations:** 1 Department of Nuclear Medicine, University of KwaZulu-Natal, Inkosi Albert Luthuli Central Hospital, Durban, South Africa

## Abstract

**Background:**

Differentiated thyroid cancer (DTC) typically carries a favourable prognosis, with 10-year survival rates between 85 and 99%. However, 20% – 30% of papillary and follicular thyroid cancers may become refractory to radioiodine therapy, resulting in thyroglobulin-elevated negative iodine scintigraphy (TENIS) syndrome. Management of radioiodine-refractory (RAIR) DTC is complex, especially in settings with limited access to tyrosine kinase inhibitors (TKIs). Alternative therapies, such as peptide receptor radionuclide therapy, have shown promise in select cases. This study aimed to identify the optimal thyroglobulin (Tg) level for predicting FDG-avid disease in TENIS syndrome using ^18^F- fluorodeoxyglucose positron emission tomography/computed tomography (FDG PET/CT) imaging.

**Methods:**

A retrospective analysis was conducted on 65 patients who had undergone total thyroidectomy and radioactive iodine ablation. Inclusion criteria were elevated Tg levels and negative iodine-131 whole-body scans (WBS), followed by ^18^F-FDG PET/CT imaging. Thyroid stimulating hormone (TSH), Tg and anti-Tg antibody levels were recorded at the time of imaging. Positron emission tomography/computed tomography scans were evaluated for abnormal uptake, and lesion locations were documented. Receiver operating characteristic (ROC) curve analysis and Youden’s index were used to determine the Tg threshold predictive of FDG-avid disease.

**Results:**

Among the 65 patients (median age 59; 76% female), 60% showed FDG-avid metastases. Cervical lymph node involvement was most common (27%), followed by lung (6%) and skeletal metastases (4%). Patients with positive PET/CT findings had significantly higher median Tg levels (79.9 ng/mL) compared to those with negative scans (34 ng/mL; *p* = 0.035). ROC analysis identified a Tg cut-off of > 15 ng/mL as optimal, yielding 60% sensitivity, 61% specificity, 74% PPV and 46% NPV.

**Conclusion:**

A Tg level above 15 ng/mL may serve as a useful threshold for identifying FDG-avid disease in TENIS syndrome. In resource-limited settings, this cut-off could guide selective use of PET/CT imaging, improving diagnostic efficiency and reducing unnecessary scans.

# Abstract: Filter freak out: How iterations and blurring affect positron emission tomography contrast for tiny targets

**Published:** 2026-07-28

**Authors:** T. Mkhize, E.V. Jonas, L. Thifhulufhelwi, A. Moodley, Z. Jwara, N. Mohamed, N. Moodley, B. Hadebe, M. Vorster

**Affiliations:** 1 Department of Nuclear Medicine, University of KwaZulu-Natal, Durban, South Africa; 2 Inkosi Albert Luthuli Central Hospital, Durban, South Africa

## Abstract

**Background:**

Reliable quantification in positron emission tomography (PET) imaging hinges on accurate contrast recovery (CR), especially for small lesions. Reconstruction parameters – including iteration count, subset number and Gaussian post-filtering – play a critical role in determining image quality and CR performance. Misadjustments can lead to underestimation or overcompensation, compromising diagnostic accuracy.

**Aim:**

This study aimed to evaluate how variations in reconstruction parameters affect CR, with a focus on small lesion detectability, using a NEMA phantom and Siemens Biograph 64 positron emission tomography/computed tomography (PET/CT) system.

**Methods:**

Phantom data were reconstructed using combinations of iterations (2, 3 and 8), Gaussian filters (4 mm, 4.5 mm and 5 mm) and a fixed 21 subsets. Contrast recovery values were calculated for six fillable spheres ranging from 10 mm to 37 mm in diameter. Metrics included SUVmax, SUVmean and SUVpeak.

**Results:**

Using two iterations with a 5-mm filter, the 13 mm sphere’s SUVpeak fell below acceptable thresholds. Reducing the filter to 4 mm led to overcompensation, with two spheres exceeding CR limits. A 4.5-mm filter slightly improved results but still failed to meet SUVpeak criteria for the 13-mm sphere. Increasing iterations to 8 with a 4.5-mm filter resulted in excessive granularity and noise, with multiple spheres surpassing SUVmax limits. A moderate setting of three iterations and 4.5 mm filtering produced more compliant SUVmean and SUVpeak values although SUVmax remained elevated and image noise persisted. These findings underscore the complexity of balancing resolution and noise across lesion sizes.

**Conclusion:**

Reconstruction parameters profoundly influence CR, particularly for lesions under 15 mm. While higher iterations and reduced filtering enhance CR, they also elevate noise and risk overestimation. An iteration-subset product near 60 (e.g. 3 × 21) may offer a practical compromise. Precision tuning is essential to avoid diagnostic pitfalls and ensure consistent image quality.

# Abstract: Healthcare workers’ awareness, accessibility and attitude towards nuclear medicine services in Lake Zone public referral hospitals, Tanzania

**Published:** 2026-07-28

**Authors:** A. Malabe, J. Mwazembeni, M. Kahts

**Affiliations:** 1 Radiopharmacy Department, Sefako Makgatho Health Sciences University, Ga-Rankuwa, South Africa

## Abstract

**Background:**

Cancer continues to pose a major global health challenge, with 19.3 million new cases and 10 million deaths reported in 2020. Projections estimate a 47% increase in cancer burden by 2040, reaching 28.4 million cases. Low- and middle-income countries, including Tanzania, account for three-quarters of cancer-related deaths, with cervical, breast and prostate cancers contributing significantly. This surge is linked to infection-related malignancies, lifestyle changes and environmental factors, placing financial strain on healthcare systems and reducing treatment success. Strengthening early detection and diagnostic capacity, particularly through nuclear medicine, is essential to address this crisis. This study aimed to evaluate healthcare workers’ awareness, accessibility and attitudes towards nuclear medicine services in public referral hospitals in Tanzania’s Lake Zone.

**Methods:**

A descriptive, cross-sectional survey was conducted among 344 healthcare professionals – including doctors, nurses, pharmacists and radiographers – from three public referral hospitals between 28 February and 31 March 2025. Participants completed a self-administered questionnaire assessing their knowledge, access and perceptions of nuclear medicine services.

**Results:**

Most respondents were female nurses aged 30–39 years, with diploma (42.2%) and degree (41.6%) qualifications. Tertiary hospitals accounted for 70.6% of participants. Those with ≥ 6 years of experience comprised 58.7%. Awareness of nuclear medicine services was high (84.6%), especially among degree holders (90.2%), while diploma holders showed lower awareness (2.1%; *p* = 0.04). Accessibility was rated as ‘somewhat accessible’ by 44.5%, with equipment and resource limitations cited. Attitudes were overwhelmingly positive (87.8%), with strong belief in the value of education and awareness to improve service utilisation.

**Conclusion:**

Healthcare workers in Tanzania’s Lake Zone demonstrate strong awareness and positive attitudes towards nuclear medicine. However, limited accessibility remains a barrier. Investment in equipment, cost reduction and integration of nuclear medicine education into early medical training are essential to enhance service delivery and cancer care outcomes.

# Abstract: Breaking boundaries in FAP-targeted therapy: Early insights from novel [Lu177] radioligands

**Published:** 2026-07-28

**Authors:** C. Kaoma, H. Ndlovu, K. Ramonaheng, J. Kabunda, K.M.G. Mokoala, A. Mdlophane, M. Sipho, M. Shungube, S. Sibiya, M.I. Qebetu, P. Goorhoo, K. Banda, L. Smith, C. Swanepoel, C. Davis, J. Jensen, N. George, I. Shoucair, I. Tunali, A. Balar, M. Axelson, M. Sathekge

**Affiliations:** 1 Nuclear Medicine Research Infrastructure, Pretoria, South Africa; 2 Eli Lilly and Company, Indianapolis, IN, United States

## Abstract

**Background:**

Fibroblast activation protein-α (FAP) is a surface glycoprotein with minimal expression in healthy adult tissues but is significantly overexpressed in cancer-associated fibroblasts across various epithelial tumours. Current FAP-targeting radioligands have been limited by rapid clearance from tumour sites, reducing their therapeutic efficacy. This Phase 0 study aimed to evaluate the tumour retention, imaging concordance and safety of three novel [Lu-177]-labelled FAP ligands – [Lu-177]-PNT3087, [Lu-177]-PNT3090 and [Lu-177]-PNT3106 – administered at subtherapeutic doses (1 GBq).

**Methods:**

This open-label, first-in-human, single-centre Phase 0 trial enrolled breast cancer patients with confirmed FAP overexpression on [Ga-68]-FAPI-46 positron emission tomography/computed tomography (PET/CT) imaging. Each participant received a 1 GBq (± 10%) dose of one ligand on Day 1, followed by a 2-week washout and a second 1 GBq (± 10%) dose of a different ligand on Day 15. Ligand sequence was based on enrolment order. Single photon emission computed tomography (SPECT)/CT imaging for dosimetry was conducted at 4–6 h, 24–30 h and 120–144 h post administration using the GE HealthCare StarGuide system.

**Results:**

Eight patients with metastatic breast cancer (HR+/HER2+ [*n* = 2], HR+/HER2– [*n* = 5], HR–/HER2+ [*n* = 1]) received 14 total doses of [Lu-177]-FAP ligands. Six patients received two ligands, while two received one. The mean administered activity was 1.03 GBq (range: 0.9–1.16 GBq). No adverse events were reported. SPECT/CT imaging demonstrated up to 144-h retention for all three ligands. Tumour-to-background contrast was visually favourable across ligands. [Lu-177]PNT3090 and [Lu-177]-PNT3106 showed stronger visual concordance with baseline PET/CT imaging than [Lu-177]-PNT3087. However, [Lu-177]-PNT3106 showed off-target uptake in the pancreas (*n* = 2) and salivary glands (*n* = 3). Enrolment is ongoing.

**Conclusion:**

The novel FAP-targeted ligands [Lu-177]-PNT3090, [Lu-177]-PNT3106 and [Lu-177]-PNT3087 exhibited excellent tumour retention and imaging concordance in FAP-expressing metastatic breast cancer. These findings support continued clinical development of these agents.

# Abstract: Lighting up the neck: Nuclear medicine in the diagnosis and treatment of a carotid body tumour

**Published:** 2026-07-28

**Authors:** L. Smith, C. Swanepoel, C. Davis

**Affiliations:** 1 Department of Nuclear Medicine, Steve Biko Academic Hospital, Pretoria, South Africa; 2 Nuclear Medicine Research Infrastructure, Pretoria, South Africa

## Abstract

**Background:**

Carotid body tumours are rare, benign tumours that arise from the carotid body. Due to the position of these tumours, biopsies are usually avoided because the tumours have very high vascularity and usually need to be surgically removed. Accurate diagnosis and effective treatment of such tumour types pose a significant challenge because of their nature of origin and placement.

**Methods:**

The management of carotid body tumours through advanced imaging techniques such as positron emission tomography/computed tomography (PET/CT) with Gallium-68 DOTATATE and targeted radionuclide therapies such as Lutetium-177 DOTATATE allows for personalised approaches and treatment, improving patient outcomes.

**Results:**

Both patients presented with swelling in the neck region. Because of the invasive nature of the procedure, neither patient underwent a biopsy. While both patients had magnetic resonance imaging (MRI) scans, the second patient also underwent CT and ultrasound imaging to assess the extent of the disease. This highlights, once again, the importance of emphasising the role of Nuclear Medicine in both the imaging and treatment of patients with carotid body tumours.

**Conclusion:**

Nuclear medicine has revolutionised not only the diagnosis but also the possibility of management of carotid body tumours by offering highly sensitive functional imaging and emerging theranostic treatment options.

# Abstract: The prognostic value of ^18^F-fluorodeoxyglucose positron emission tomography/computed tomography metrics in lung cancer

**Published:** 2026-07-28

**Authors:** T.S. Nxasana, N.D. Ndlovu, L.M. Harry, L. Gabela, S.N. Madi, S. Masikane, M. Patel, V. Pillay, B.P. Hadebe, M. Vorster

**Affiliations:** 1 Department of Nuclear Medicine, University of KwaZulu-Natal, Durban, South Africa; 2 Inkosi Albert Luthuli Central Hospital, Durban, South Africa; 3 Victoria Mxenge Hospital, Durban, South Africa

## Abstract

**Background:**

Lung cancer remains one of the most prevalent and deadly cancers worldwide. While ^18^F- fluorodeoxyglucose positron emission tomography/computed tomography (FDG PET/CT) is routinely used for staging, its prognostic utility – particularly pretreatment metrics such as metabolic tumour volume (MTV) and total lesion glycolysis (TLG) – remains debated. This study aimed to evaluate the predictive value of PET/CT parameters in assessing survival and treatment response in therapy-naive lung cancer patients.

**Methods:**

A retrospective analysis was conducted on 88 patients with histologically confirmed lung cancer who underwent ^18^F-FDG PET/CT prior to treatment. Semi-automatic spherical volumes of interest (VOIs) were drawn around primary lesions using a standardised uptake value (SUV) threshold of 1.4 and a 3D iso-contour of 40%. Metrics including SUVmax, SUVmean, MTV and TLG were correlated with progression-free survival (PFS) and overall survival (OS). Statistical analysis was performed using R software (v3.6.3), and Kaplan–Meier survival curves were generated. Cut-off values were set at > 9 for SUVmax, > 3 for SUVmean, > 500 for MTV and > 1000 for TLG.

**Results:**

The cohort included 31 females and 57 males, with a median age of 63 years. Adenocarcinoma was the most common subtype (68%). Most patients (89%) had died by the time of analysis. SUVmax was higher in deceased patients (median 12.2) compared to survivors (10.8) although not statistically significant (*p* = 0.563). Total lesion glycolysis and MTV were significantly elevated in deceased patients: TLG median 616 vs. 87.8 (*p* = 0.007) and MTV median 72.4 vs. 12.5 (*p* = 0.001). A TLG > 1000 was strongly associated with poorer survival.

**Conclusion:**

Higher pretreatment PET/CT metrics – particularly TLG and MTV – correlate with worse survival outcomes in lung cancer. TLG > 1000 may serve as a prognostic marker, supporting its role in guiding treatment strategies and risk stratification.

# Abstract: Should ^18^F-FDG PET/CT replace ^99m^Tc-MDP bone scintigraphy in the management of breast cancer patients with suspected skeletal metastases?

**Published:** 2026-07-28

**Authors:** M. Patel, L.M. Harry, T.S. Nxasana, N.D. Ndlovu, L. Gabela, S. Masikane, V. Pillay, B. Hadebe, M. Vorster

**Affiliations:** 1 Department of Nuclear Medicine, University of KwaZulu-Natal, Durban, South Africa; 2 Inkosi Albert Luthuli Central Hospital, Durban, South Africa; 3 Victoria Mxenge Hospital, Durban, South Africa

## Abstract

**Background:**

Breast cancer is the leading cause of cancer-related mortality in women and frequently metastasises to bone. Early and accurate detection of skeletal metastases is critical for staging, treatment planning, monitoring and prognosis. In South Africa, ^99m^Tc-MDP bone scintigraphy is commonly used because of cost and accessibility although ^18^F-fluorodeoxyglucose positron emission tomography/computed tomography (FDG PET/CT) offers superior sensitivity for detecting lytic lesions, marrow involvement and soft tissue metastases. This study compares the diagnostic yield and clinical impact of both modalities to assess the comparative effectiveness of ^99m^Tc-MDP bone scintigraphy and ^18^F-FDG PET/CT in detecting skeletal metastases and influencing staging and management in breast cancer patients.

**Methods:**

A cross-sectional analysis was conducted on 100 breast cancer patients who underwent both imaging modalities within 42 days between January 2017 and September 2024. Lesion-by-lesion comparisons were made, and changes in staging and management were documented.

**Results:**

The median age was 55 years. Histological subtypes included invasive ductal carcinoma (49%), invasive breast cancer no special type (NST) (45%) and invasive lobular carcinoma (4%). Tumour staging revealed 53% with T4 lesions. Positron emission tomography/computed tomography detected 421 lesions versus 243 on bone scintigraphy. Positron emission tomography/computed tomography led to staging and management changes in 27 patients, compared to 22 with bone scintigraphy. Positron emission tomography/computed tomography uniquely identified bone lesions in 10 patients and soft tissue metastases – including lung, liver and lymph nodes – in several cases, resulting in additional staging upgrades. Bone scintigraphy detected lesions missed by PET/CT in five patients, affecting management in three.

**Conclusion:**

^18^F-FDG PET/CT demonstrated superior lesion detection and broader metastatic assessment, significantly impacting staging and treatment decisions. Given its diagnostic advantages, PET/CT should replace bone scintigraphy in breast cancer management where feasible.

# Abstract: Can prostate-specific membrane antigen positron emission tomography tumour-to-salivary gland ratio to predict response to ^177^Lu-PSMA and ^225^Ac-PSMA radioligand therapy

**Published:** 2026-07-28

**Authors:** L. Gabela, L.M. Harry, T.S. Nxasana, N.D. Ndlovu, T.D Mkhize, V. Pillay, S. Masikane, M. Patel, N. Madi, P.G. Chiumia, C.A. Kalowa, B.P. Hadebe, M. Vorster

**Affiliations:** 1 Department of Nuclear Medicine, University of KwaZulu-Natal, Durban, South Africa; 2 Inkosi Albert Luthuli Central Hospital, Durban, South Africa

## Abstract

**Background:**

Prostate-specific membrane antigen (PSMA) is a transmembrane glycoprotein highly expressed on prostate cancer cells, particularly in advanced metastatic castration-resistant prostate cancer (mCRPC). Prior to radioligand therapy with ^177^Lu-PSMA or ^225^Ac-PSMA, ^68^Ga-PSMA positron emission tomography/computed tomography (PET/CT) is used to assess PSMA expression. However, patient responses vary. Previous studies suggest that the tumour-to-salivary gland ratio may predict prostate-specific antigen (PSA) response and overall survival.

This study aimed to determine whether the quantitative positron emission tomography (PET) tumour-to-salivary gland ratio (qPSG score) can predict treatment response and survival outcomes in mCRPC patients undergoing PSMA-targeted therapy.

**Methods:**

A retrospective analysis was conducted on 45 mCRPC patients treated with ^177^Lu-PSMA and/or ^225^Ac-PSMA. Baseline ^68^Ga-PSMA PET/CT scans were used to calculate qPSG scores, defined as the SUVmean ratio of the most avid tumour lesion to the parotid gland. Patients were grouped into high (qPSG > 1.5) and low (qPSG ≤ 1.5) categories. Outcomes included PSA decline ≥ 50% and overall survival (OS). Disease sites were classified as bone only, bone + lymph, lymph only or other.

**Results:**

Forty-five prostate cancer patients (mean age: 66.4 years) undergoing PSMA-targeted therapy were retrospectively analysed. PSA-response (≥ 50% decline) was observed in 55.6% of patients. Response rates were similar between the High (qPSG > 1.5) and Low (qPSG ≤ 1.5) groups (57.9% vs 58.3%, *p* = 1.00). Survival outcomes were also comparable (*p* = 0.82) although more deaths occurred in the low group (qPSG score ≤ 1.5).

Disease site was categorised as bone only, bone + lymph, lymph only or other. Patients with lymph node–only disease showed better survival, while those with bone involvement had higher mortality although not statistically significant (*p* = 0.20).

**Conclusion:**

While lower qPSG scores and bone involvement showed a trend towards poorer outcomes, statistical significance was not reached. The qPSG score may hold prognostic value, but larger studies are needed to confirm its utility in guiding PSMA-targeted therapy selection.

# Abstract: The efficacy of [^68^Ga]Ga-NODAGA^ZOL^ positron emission tomography/computed tomography imaging in detecting bone metastases in patients diagnosed with breast cancer

**Published:** 2026-07-28

**Authors:** C. Davis

**Affiliations:** 1 Nuclear Medicine Research Infrastructure, Pretoria, South Africa; 2 Steve Biko Academic Hospital, Pretoria, South Africa

## Abstract

**Background:**

Breast and prostate cancers have a high propensity for skeletal metastases, commonly affecting the ribs, pelvis, spine and long bones. If undetected, bone metastases can result in serious complications such as pathological fractures, hypercalcaemia and spinal cord compression. Accurate imaging during staging and re-staging is essential for effective treatment planning and prognosis. Traditionally, [^99m^Tc]Tc-MDP bone scintigraphy is the standard imaging modality for detecting bone metastases. However, positron emission tomography/computed tomography (PET/CT) offers superior spatial resolution, and ^68^Gallium has emerged as a promising tracer because of its favourable physical properties and generator availability. Zoledronate, a bisphosphonate with high affinity for hydroxyapatite, has been conjugated with DOTA and NODAGA to form DOTA^ZOL^ and NODAGA^ZOL^, respectively, and labelled with ^68^Ga for PET imaging. While [^68^Ga]Ga-NODAGA^ZOL^ has shown efficacy in prostate cancer, its utility in breast cancer remains unexplored.

**Method:**

Ten female breast cancer patients underwent [^68^Ga]Ga-NODAGA^ZOL^ PET/CT scans because of the unavailability of [^99m^Tc]Tc-MDP. Each received an average intravenous dose of 2.82mCi. Whole-body PET/CT imaging was performed 60 min post-injection, with a scan duration of approximately 20 min. A non-diagnostic CT was used for attenuation correction and anatomical reference.

**Results:**

All scans were of high quality with favourable target-to-background ratios. Findings included maxillary inflammation, osteo-degenerative changes in vertebral bodies, facet and shoulder joints and confirmed osseous metastases.

**Conclusion:**

[^68^Ga]Ga-NODAGA^ZOL^ PET/CT demonstrated potential as a viable alternative to [^99m^Tc]Tc-MDP bone scintigraphy for staging and re-staging in breast cancer patients. Its diagnostic performance supports further investigation into its broader clinical application in oncology imaging.

# Abstract: The role of Tc-99m HMPAO SPECT imaging in evaluating cerebral perfusion after revascularisation in patients with moyamoya disease

**Published:** 2026-07-28

**Authors:** K. Joseph, F. Smith, K. Hlongwa, S. More

**Affiliations:** 1 Divison of Nuclear Medicine, Department of Radiation Medicine, University of Cape Town, Cape Town, South Africa; 2 Red Cross War Memorial Children’s Hospital, Cape Town, South Africa

## Abstract

**Background:**

Moyamoya disease (MMD) is a rare cerebrovascular condition marked by progressive narrowing of the internal carotid arteries and their branches, resulting in diminished cerebral perfusion. On angiography, this vascular pattern resembles a ‘puff of smoke’, which inspired the disease’s name. While magnetic resonance imaging (MRI) is the standard imaging modality for diagnosis and follow-up, Tc-99m HMPAO single photon emission computed tomography (SPECT) offers complementary insights into cerebral blood flow and perfusion dynamics. This study explores the utility of Tc-99m HMPAO SPECT in evaluating cerebral perfusion changes following surgical revascularisation in MMD patients.

**Methods:**

A retrospective review was conducted on six patients diagnosed with MMD who underwent both Tc-99m HMPAO SPECT and MRI imaging before and after revascularisation. Patient records from 2004 to 2024 were analysed to assess changes in cerebral perfusion and vascular morphology. The primary outcome was the correlation between perfusion changes on SPECT and anatomical changes on MRI post-surgery.

**Results:**

Of the six patients, two showed improved cerebral perfusion post-revascularisation, particularly in the cerebral cortex and left parietal-occipital regions. Four patients exhibited disease progression. In three cases, SPECT findings aligned with MRI results, confirming both perfusion improvement and anatomical changes. However, in the remaining three, perfusion changes observed on SPECT did not correspond with MRI findings, highlighting the complexity of interpreting cerebral haemodynamics. Tc-99m HMPAO SPECT proved sensitive to metabolic shifts and regional blood flow reductions, offering valuable insights beyond structural imaging.

**Conclusion:**

Tc-99m HMPAO SPECT imaging demonstrates significant potential in assessing revascularisation outcomes in MMD patients. Its ability to detect perfusion changes complements MRI and enhances understanding of cerebral haemodynamics. These findings support the integration of SPECT into routine post-surgical evaluation protocols for MMD.

# Abstract: Theranostic approach and clinical internal dosimetry implementation

**Published:** 2026-07-28

**Authors:** S.E. Bouyoucef

**Affiliations:** 1 Service de Médecine Nucléaire CHU Bab El Oued, Bab El Oued, Algeria

## Abstract

**Backgrounds:**

In nuclear medicine, the distribution of administered radiopharmaceuticals for diagnostic and therapeutic purposes is central to internal dosimetry. Accurate dosimetry is especially critical for beta and alpha particle emitters to optimise treatment efficacy and minimise toxicity. With the emergence of validated software tools, the routine implementation of internal dosimetry is becoming increasingly feasible. This study aims to demonstrate the clinical value of internal dosimetry in: (1) Evaluating [^177^Lu]Lu-PSMA therapy for metastatic prostate cancer and Iodine-131 therapy for metastatic thyroid cancer; (2) Calculating precise pre- and post-treatment absorbed doses to maximise therapeutic impact; (3) Estimating radiation exposure to organs at risk (OAR), including kidneys, salivary and lacrimal glands for ^177^Lu-PSMA and bone marrow, residual thyroid and salivary glands for I-131.

**Methods:**

Absorbed dose calculations were performed using the medical internal radiation dose (MIRD) schema, implemented via OLINDA/EXM software. Predictive dosimetry was based on positron emission tomography (PET) imaging at 15 min and 1 h post [^68^Ga]PSMA administration. single photon emission computed tomography (SPECT)/CT calibration used 1.4 GBq of ^177^Lu-PSMA and 111 MBq of I-131. Six doses of ^177^Lu-PSMA (7.7 GBq each) were administered to three patients. Imaging included whole-body scans and three-bed SPECT/CT at 1 h, 48 h and 72 h post-injection.

**Results:**

Dosimetry revealed right kidney doses of 2.8 mSv and left kidney doses of 3.2 mSv. Parotid and salivary glands received 18.6 Gy and 13.8 Gy over four doses, respectively. Kidney function remained normal. In thyroid cancer patients, poor I-131 response correlated with low lesion avidity. Whole-body retention was < 50 mCi at 48 h, and salivary gland dose remained < 10 Gy for a 200 mCi administration.

**Conclusion:**

Integrating internal dosimetry into nuclear medicine practice enhances predictive accuracy for radionuclide therapy, supporting individualised treatment planning and improved patient outcomes.

# Abstract: The impact of [Lu]Lu-177 DOTATATE in the treatment of neuroendocrine tumours in people living with HIV: A case report

**Published:** 2026-07-28

**Authors:** T. Mefane, A. Brink, K. Hlongwa, N. Jacobs, A. AL Marrani, P. Rivombo, S. More

**Affiliations:** 1 Divison of Nuclear Medicine, Department of Radiation Medicine, University of Cape Town, Cape Town, South Africa

## Abstract

**Background:**

Peptide receptor radionuclide therapy (PRRT) has transitioned from research to routine clinical use for treating neuroendocrine neoplasms (NENs). However, its effectiveness in immunosuppressed patients, particularly those living with human immunodeficiency virus (HIV), remains underexplored.

**Methods:**

A 48-year-old HIV-positive female, virally suppressed but with a persistently low CD4 count (< 200 cells/mL), presented with an 8-month history of upper gastrointestinal bleeding. She was also diabetic, managed with metformin. Imaging and histology confirmed a grade II neuroendocrine tumour of the Ampulla of Vater with liver metastases, rendering the disease inoperable. She had an ECOG performance status of 1 and was referred for PRRT.

She received nine cycles of [^177^Lu]Lu-DOTATATE therapy totalling 64.01 GBq, administered over two phases: the first between December 2021 and August 2022 and the second from March to August 2024. Treatment was intermittently delayed because of recurrent GI bleeding requiring transfusions. During therapy, her CD4 count declined further and viral load increased, raising concerns about PRRT’s impact on immunosuppression. Despite adherence counselling and prophylactic measures, her CD4 count remained low.

One year into therapy, she achieved stable disease. However, post-treatment complications included mild bone marrow suppression and persistent thrombocytopenia. Bone marrow aspirate ruled out tumour infiltration, suggesting PRRT or HIV-related immunosuppression as potential causes. Her condition deteriorated, and she eventually succumbed to the disease.

**Results:**

This case underscores the importance of optimising antiretroviral therapy, monitoring CD4 counts and providing prophylaxis against opportunistic infections in HIV-positive patients undergoing PRRT. Despite immunosuppression, the patient showed initial therapeutic response without opportunistic infections.

**Conclusion:**

Peptide receptor radionuclide therapy can be effective in immunosuppressed PLHIV, but further research is needed to refine treatment protocols and assess long-term safety and efficacy in this vulnerable population.

# Abstract: A comparison of ^18^F-fluorodeoxyglucose positron emission tomography/computed tomography and MRI in aiding the diagnosis of Parkinson’s disease

**Published:** 2026-07-28

**Authors:** L.M. Harry, A.P. Msomi, N.D. Ndlovu, L. Gabela, S. Masikane, L. Nxasana, M. Patel, V. Pillay, B.P. Hadebe, L. Amusa, R. Perumal, M. Vorster

**Affiliations:** 1 Department of Nuclear Medicine, University of KwaZulu-Natal, Durban, South Africa; 2 Department of Radiology, University of KwaZulu-Natal, Durban, South Africa; 3 Department of Pulmonology, University of KwaZulu-Natal, Durban, South Africa

## Abstract

**Background:**

Neurodegenerative diseases, particularly Parkinson’s and Alzheimer’s, are increasingly prevalent and impose significant burdens on families and healthcare systems. In Parkinson’s disease (PD), metabolic changes often precede structural abnormalities, making functional imaging crucial. While magnetic resonance imaging (MRI) is widely used to exclude anatomical causes, it lacks specificity in differentiating Parkinsonian subtypes. ^18^F-fluorodeoxyglucose positron emission tomography/computed tomography (FDG PET/CT), which assesses regional cerebral glucose metabolism, offers distinct functional insights and may enhance diagnostic accuracy when combined with MRI. This study aimed to compare the diagnostic performance of ^18^F-FDG PET/CT and MRI in identifying Parkinson’s disease and related neurodegenerative disorders.

**Methods:**

Ninety-seven patients with suspected PD underwent both ^18^F-FDG PET/CT and MRI. A nuclear medicine physician and a radiologist independently reviewed the scans, blinded to each other’s interpretations and the final clinical diagnosis. Each modality was assessed qualitatively and categorised as ‘Yes’, ‘No’ or ‘Maybe’ for PD. The final diagnosis, determined by a neurologist through a comprehensive clinical evaluation, served as the gold standard. Agreement between modalities and clinical consensus was measured using Cohen’s Kappa coefficient.

**Results:**

Positron emission tomography/computed tomography showed moderate agreement with clinical consensus (*κ* = 0.520), outperforming MRI, which showed poor agreement (*κ* = 0.081). Positron emission tomography/computed tomography accurately identified 96% of idiopathic PD cases, 67% of multiple system atrophy (MSA), 100% of progressive supranuclear palsy (PSP) and 100% of dementia with Lewy bodies (DLB). MRI detected only 2% of idiopathic PD and had limited sensitivity for other subtypes. Positron emission tomography/computed tomography also misclassified 88% of normal cases, indicating high sensitivity but lower specificity.

**Conclusion:**

^18^F-FDG PET/CT provides superior functional insights and diagnostic alignment with clinical consensus compared to MRI, particularly in differentiating Parkinsonian subtypes. It is a valuable tool for enhancing diagnostic accuracy in neurodegenerative disease evaluation.

# Abstract: Feasibility and diagnostic accuracy of reducing milk scan acquisition time to 30 min at a tertiary state hospital in South Africa

**Published:** 2026-07-28

**Authors:** N.B. Mlaba, N.E. Nyakale, A.B. Rahmani

**Affiliations:** 1 Nuclear Medicine Department, Sefako Makgatho Health Sciences University, Ga-Rankuwa, South Africa; 2 Dr George Mukhari Academic Hospital, Ga-Rankuwa, South Africa

## Abstract

**Background:**

Gastroesophageal reflux (GER) is a common, typically self-limiting condition in infants and children. However, when GER progresses to gastroesophageal reflux disease (GERD), it can result in serious complications such as poor weight gain, failure to thrive, oesophagitis and recurrent respiratory infections. Early and accurate diagnosis is essential to guide appropriate management and prevent long-term morbidity.

Gastroesophageal reflux scintigraphy (GERS), also known as the milk scan, is a valuable nuclear medicine technique for detecting reflux and aspiration. Despite its clinical utility, there is no universally accepted imaging protocol. The standard 60-min acquisition period is designed to capture sufficient diagnostic data but may be difficult for paediatric patients because of discomfort and movement, which can compromise image quality. A shorter acquisition time could improve patient compliance and reduce motion artefacts, provided diagnostic accuracy is maintained.

This study aimed to retrospectively evaluate whether a 30-min acquisition period yields comparable diagnostic information to the conventional 60-min protocol in paediatric patients referred for GERS at Dr George Mukhari Academic Hospital (DGMAH).

**Methods:**

A retrospective review was conducted on paediatric patients who underwent GERS for suspected GERD between January 2021 and December 2024. Imaging data were analysed for both the full 60-min acquisition and he initial 30-min period. Diagnostic outcomes were compared.

**Results:**

Among 71 patients (median age: 3 months), 92.5% of positive findings were detected within the first 30 min. Only 7.5% required the full 60-min scan for diagnosis (*p* < 0.001). Motion artefacts were less frequent in the first 30 min (7.1%) compared to the latter half (11.3%).

**Conclusion:**

A 30-min acquisition protocol provides statistically comparable diagnostic accuracy to the standard 60-min scan and may enhance patient comfort and workflow efficiency. Further multicentre validation is recommended.

# Abstract: Novel positron emission tomography evaluation of Nectin-4 expression with [^68^Ga]Ga-AKY-1189 in urothelial bladder cancer

**Published:** 2026-07-28

**Authors:** J. Kabunda, H. Ndlovu, A. Mdlophane, K. Ramonaheng, S. Mdanda, M. Shungube, Y. Mzizi, M. Qebetu, L. Smith, R. Sebatana, C. Swanepoel, C. Davis, S. Sibiya, K. Nasr, S. More, M. Vorster, K. Mokoala, T. Benedum, J. Horton, S. Ron-Bigger, A. Czibere, J. Kovacs, K. Herrmann, M.M. Sathekge

**Affiliations:** 1 Nuclear Medicine Research infrastructure (NuMeRI), Department of Nuclear Medicine, University of Pretoria and Steve Biko Academic Hospital, Pretoria, South Africa; 2 AKTIS Oncology, Boston, United States; 3 Department of Nuclear Medicine, University of Cape Town, Cape Town, South Africa; 4 Department of Nuclear Medicine, University of KwaZulu-Natal, Durban, South Africa

## Abstract

**Background:**

Nectin-4 is a cell-surface protein overexpressed in up to 90% of urothelial bladder cancers (UBC), making it a compelling therapeutic target. Enfortumab vedotin, an antibody–drug conjugate directed against Nectin-4, has demonstrated clinical efficacy in advanced UBC. This highlights the need for non-invasive imaging tools to identify Nectin-4–expressing tumours and guide treatment decisions. [^68^Ga]Ga-AKY-1189 is a novel radiolabelled miniprotein developed to target Nectin-4. This study aimed to assess its safety and tumour-targeting capability using positron emission tomography/computed tomography (PET/CT) imaging in patients with metastatic UBC.

**Methods:**

Eight patients with locally advanced or metastatic UBC, previously referred for [^18^F]FDG PET/CT, underwent [^68^Ga]Ga-AKY-1189 PET/CT. Imaging was performed 1 h post-injection (mean activity: 4.65 mCi). Tracer uptake was scored from 0 to 3, with scores ≥ 2 considered positive. SUVmax and SUVmean values were recorded for primary and metastatic lesions.

**Results:**

[^68^Ga]Ga-AKY-1189 was well tolerated in all patients. Tracer uptake was observed in all known tumour sites. Physiological distribution included salivary and lacrimal glands, nasal mucosa, gastrointestinal tract, pancreas, prostate and urinary excretion. Uptake was observed in primary tumours (6 patients), lymph nodes (4), visceral metastases (3) and skeletal metastases (1), aligning with conventional imaging. The highest uptake (Grade 3) was seen in primary and skeletal lesions. Median SUVmax values were: primary (20.68), lymph nodes (15.42), visceral (23.76) and skeletal (37.26). Median SUVmean values were: primary (12.13), lymph nodes (3.97), visceral (7.69) and skeletal (10.09).

**Conclusion:**

[^68^Ga]Ga-AKY-1189 PET/CT demonstrates strong potential as a non-invasive imaging biomarker for Nectin-4 expression in UBC. It may aid in selecting candidates for Nectin-4–targeted therapies and monitoring treatment response, offering a valuable tool for personalised oncology care.

# Abstract: Metabolic parameters and disease burden in Rosai-Dorfman disease and Juvenile Xanthogranuloma: A comparative positron emission tomography/computed tomography case analysis

**Published:** 2026-07-28

**Authors:** K. Hlongwa, F. Smith, S. More

**Affiliations:** 1 Divison of Nuclear Medicine, Department of Radiation Medicine, University of Cape Town, Cape Town, South Africa; 2 Red Cross War Memorial Children’s Hospital, Cape Town, South Africa

## Abstract

**Background:**

Rosai-Dorfman disease (RDD) and juvenile xanthogranuloma (JXG) are rare non-Langerhans cell histiocytic disorders predominantly affecting children. While ^18^F-fluorodeoxyglucose positron emission tomography/computed tomography (FDG PET/CT) is routinely used for staging and assessing disease extent, limited data exist on how metabolic activity correlates with disease burden in these conditions. This study aimed to compare quantitative metabolic parameters and overall disease burden between paediatric patients with RDD and JXG using ^18^F-FDG PET/CT imaging.

**Methods:**

Staging PET/CT scans from six paediatric patients,: three with RDD and three with JXG, were retrospectively analysed. For each lesion, SUVmax, SUVmean, SUVpeak, metabolic tumour volume (MTV) and total lesion glycolysis (TLG) were calculated. Whole-body metrics for SUVmax, SUVmean, SUVpeak, MTV and TLG were also assessed.

**Results:**

Rosai-Dorfman disease lesions demonstrated notably higher metabolic activity than JXG. Median and mean SUVmax for RDD were 8.52 and 8.00, respectively, compared to 3.05 and 3.56 for JXG. SUVmean and SUVpeak values followed similar trends. RDD also showed elevated lesion-level MTV (mean: 77 cm^3^) and TLG (mean: 87.08), compared to JXG (MTV mean: 8.71 cm^3^; TLG mean: 13.67).

Whole-body metrics further highlighted the disparity. RDD patients had higher median and mean whole-body SUVmax (45.76 and 40.61), SUVmean (22.61 and 15.89) and SUVpeak (37.14 and 30.38). Whole-body MTV and TLG were also greater in RDD (MTV mean: 109.40 cm^3^; TLG mean: 1842.98) than in JXG (MTV mean: 46.27 cm^3^; TLG mean: 1243.08).

**Conclusion:**

Rosai-Dorfman disease exhibits significantly higher metabolic activity and disease burden than JXG, which may reflect underlying biological differences. These findings support the use of ^18^F-FDG PET/CT in guiding treatment planning and monitoring in paediatric histiocytic disorders. Further studies are warranted to validate these observations.

# Abstract: Development of quality control procedures for various [^225^Ac] PSMA injections produced at NuMeRI

**Published:** 2026-07-28

**Authors:** A. Mokoena, Y. Mzizi, H. Ndlovu, M. Shungube, S. Sibiya, A. Mdlophane, M. Qebethu, K. Ramonaheng, M. Sathekge, S. Mdanda

**Affiliations:** 1 Department of Nuclear Medicine, University of Pretoria and Steve Biko Academic Hospital, Pretoria, South Africa; 2 Nuclear Medicine Research Infrastructure (NuMeRI), Steve Biko Academic Hospital, Pretoria, South Africa

## Abstract

**Background:**

Actinium-225 labelled PSMA (^225^Ac-PSMA) compounds, including ^225^Ac-PSMA-617 trifluoroacetic acid (TFA) and ^225^Ac-PSMA-I&T, have shown promise as targeted therapies for castration-resistant prostate cancer. As clinical applications expand, there is a growing need for robust, standardised quality control (QC) protocols to ensure safety, efficacy and regulatory compliance, particularly with South African Health Products Regulatory Authority (SAHPRA) guidelines. This study aimed to develop and validate QC procedures tailored to various ^225^Ac-PSMA formulations, focusing on radionuclidic identity and purity, endotoxin levels, radiochemical purity and product stability. Analytical methods included high-performance liquid chromatography (HPLC), gamma spectrometry and thin-layer chromatography (TLC), applied across multiple production batches to assess reproducibility and reliability.

**Methods:**

^225^Ac-PSMA compounds were manually radiolabelled in a validated cleanroom environment. Production adhered to Good Radiopharmacy Practice (GRPP) and Good Manufacturing Practice (GMP) standards for investigational radiopharmaceuticals. Solutions and buffers were prepared in a Grade C area and sterilised under Grade A laminar airflow. Quality assurance included daily logging of ^225^Ac activity and risk-based process monitoring. QC testing followed EU Pharmacopoeia standards, with sterility assessed after ten half-lives of ^225^Ac (~ 100 days). Radiolabelling protocols were adapted from Kratochwil et al., 2016.

**Results:**

All ^225^Ac-PSMA batches demonstrated consistent radiochemical purity (95% – 99%), with physicochemical properties within specification. Sterility and endotoxin levels met required standards, supported by successful filter integrity tests. Radionuclidic identity was confirmed via spectral analysis although half-life-based verification posed challenges because of instrument variability.

**Conclusion:**

^225^Ac-PSMA radiopharmaceuticals were reliably produced with consistent QC outcomes across peptide variants. Implementation of validated QC protocols in a hospital cleanroom setting supports safe and compliant use in phase 1 clinical trials, reinforcing the feasibility of integrating these therapies into routine clinical practice.

# Abstract: Optimising Lu-177 SPECT quantification on a cadmium zinc telluride Gamma camera: Reconstruction parameter tuning and evaluation of single-time-point dosimetry

**Published:** 2026-07-28

**Authors:** K. Banda, M. Qebetu, T. Moeng, M. Legodi, P. Goorhoo, C. Davis, L. Smith, A. Mdlophane, S. Sibiya, H. Ndlovu, J. Kabunda, M. Sathekge, K. Ramonaheng

**Affiliations:** 1 Nuclear Medicine Research Infrastructure (NuMeRI), Pretoria, South Africa; 2 Department of Nuclear Medicine, University of Pretoria, Pretoria, South Africa; 3 Steve Biko Academic Hospital, Pretoria, South Africa

## Abstract

**Background:**

Precise activity quantification is critical for effective dosimetry in Lutetium-177 (Lu-177) radiopharmaceutical therapy (RPT), enabling accurate dose estimation and personalised treatment planning. This study investigates how single photon emission computed tomography (SPECT) image reconstruction parameters – specifically iterations and subsets – impact quantification accuracy using the StarGuide cadmium zinc telluride (CZT) gamma camera. In addition, it explores whether single-time-point (STP) dosimetry at 144 h can serve as a reliable alternative to the conventional multiple-time-point (MTP) protocol, potentially simplifying clinical workflows.

**Methods:**

A NEMA IEC body phantom with six spheres filled with Lu-177 (1.9 MBq/mL) was scanned using the GE StarGuide CZT SPECT/CT system. Imaging incorporated dual-energy windows (113 keV and 208 keV), triple-energy window scatter correction, CT-based attenuation correction and resolution recovery. Reconstructions were performed using Q.Clear and ordered subset expectation maximisation (OSEM) algorithms, with iterations ranging from 2 to 64 (10 subsets). A Lu-177 calibration factor was applied post-reconstruction. Volumes of interest (VOIs) were defined to assess activity recovery, recovery coefficients (RCs) and image noise via coefficient of variation (COV). Optimised parameters were applied to clinical data from two patients (~ 7.4 GBq Lu-177 administered), comparing STP (144 h) and MTP (4 h, 24 h, 144 h) dosimetry using MIM SurePlan MRT.

**Results:**

OSEM with 12 iterations and 10 subsets yielded optimal quantification (RC = 0.90, COV = 16.32%), with only 9.6% deviation from true activity. Default settings underestimated absorbed doses by up to 17.4%. Optimised reconstruction reduced discrepancies to < 10% for kidneys. Single-time-point dosimetry showed deviations of 38.3% and 24.4% in Patients A and B, respectively, compared to MTP.

**Conclusion:**

Optimising reconstruction parameters significantly enhances Lu-177 SPECT quantification. STP dosimetry at 144 h, when paired with optimised reconstruction, offers a promising, efficient alternative to MTP protocols for personalised RPT.

# Abstract: Comparative evaluation of image quality in high- and standard-sensitivity positron emission tomography/computed tomography systems: A phantom-based assessment

**Published:** 2026-07-28

**Authors:** K. Legodi, M. Qebetu, K. Banda, P. Goorhoo, C. Davis, L. Smith, H. Ndlovu, J. Kabunda, A. Mdlophane, S. Mdanda, S. Sibiya, M. Sathekge, K. Ramonaheng

**Affiliations:** 1 Nuclear Medicine Research Infrastructure (NuMeRI), Pretoria, South Africa; 2 Department of Nuclear Medicine, University of Pretoria, Pretoria, South Africa; 3 Steve Biko Academic Hospital, Pretoria, South Africa

## Abstract

**Background:**

Advancements in positron emission tomography/computed tomography (PET/CT) technology, such as extended axial field-of-view (AFOV) and increased system sensitivity, have led to improved image quality and reduced scan times. Despite these improvements, challenges remain with spatial resolution and signal-to-noise ratio (SNR). This study compares two PET/CT systems – Siemens Biograph Vision 450 and Vision 600 – using phantom-based analysis to evaluate image quality, contrast metrics and the feasibility of reducing administered activity without compromising diagnostic accuracy.

**Methods:**

A Jaszczak phantom with hot cylinders and cold spheres was scanned on both systems. The Vision 450 used 364.82 MBq of Ga-68, while the Vision 600 used 191.51 MBq, representing a ~ 50% dose reduction. Metrics assessed included hot/cold contrast, contrast recovery coefficient (CRC), contrast-to-noise ratio (CNR), SNR, coefficient of variation (COV) and background variability (BV).

**Results:**

Both systems produced high-quality images with distinct strengths. The Vision 600 excelled in sharpness and SNR for small spheres (8 mm and 16 mm), demonstrating strong performance in detecting small lesions at lower activity levels. The Vision 450 showed superior CNR, especially for larger spheres, with a peak CNR of 392.32 for the 25-mm hot cylinder versus 322.85 on the Vision 600. It also achieved better cold contrast recovery for larger spheres. Vision 600 had lower COV, indicating reduced image noise, although it showed slightly higher BV. The CRC analysis revealed Vision 600 was better for small lesion characterisation, while Vision 450 was more effective for larger lesions.

**Conclusion:**

Both PET/CT systems offer excellent performance, with Vision 600 supporting dose-efficient imaging of small structures and Vision 450 providing enhanced contrast for larger lesions. These insights can guide system selection and protocol optimisation based on specific clinical needs, balancing sensitivity, contrast and noise.

# Abstract: From hyperthyroidism to hidden malignancy: Incidental papillary thyroid carcinoma in Grave’s disease at Chris Hani Baragwanath Academic Hospital

**Published:** 2026-07-28

**Authors:** K.J. Kobela, M.N.B. Cossie, A. Akinwale

**Affiliations:** 1 Department of Nuclear Medicine and Molecular Imaging, University of the Witwatersrand, Johannesburg, South Africa

## Abstract

**Background:**

Graves’ disease, the most common cause of hyperthyroidism, has been shown to be associated with papillary thyroid carcinoma (PTC). The linking pathogenesis has not yet been established and is still under-investigation. We report a case of a Chris Hani Baragwanath Hospital patient diagnosed with Grave’s disease with exophthalmos, resistant to medical therapy and subsequently underwent total thyroidectomy, which revealed PTC.

**Methods:**

A 54-year-old woman, clinically presented with tremors, proximal myopathy, lid lag and exophthalmos and hyperthyroidism confirmed on biochemistry. She had positive thyroid stimulating hormone (TSH) receptor antibodies and was diagnosed with Graves’ disease.

**Results:**

A Tc-99m pertechnetate scan showed uniformly enlarged lobes with extension to the sternal notch marker, no prominent nodules and increased thyroid to background ratio with overall impression supporting clinical diagnosis of Graves’. She was resistant to Neomercazole, which she was started on since 2019 and subsequently underwent total thyroidectomy. Histology confirmed a unifocal differentiated high-grade follicular variant of papillary thyroid carcinoma (pT1 pNx) with background morphological changes of Grave’s disease.

**Conclusions:**

There is evidence showing an association between Graves’ disease and papillary thyroid carcinoma, even though the pathogenesis has not been fully elucidated. Although most cases have been found to be early-stage cancer, a meta-analysis also demonstrated a statistically significant increased risk of multifocality and distant metastasis at the time of cancer diagnosis in differentiated thyroid cancer (DTC) patients with Graves’ disease (GD) than those without GD. Further studies are needed to aid in the development of risk assessment for papillary thyroid carcinoma in patients with Grave’s disease. The prevalence and clinical significance of incidental thyroid carcinoma are worth considering when deciding on management strategies (medical or surgical) for patients with Graves’ disease, especially in the presence of thyroid nodules.

# Abstract: Discordant imaging findings in a paediatric renal transplant recipient with suspected vascular thrombosis: A case report

**Published:** 2026-07-28

**Authors:** A. AL-Marrani, K. Hlongwa, N. Jacobs, T. Mefane, P. Rivombo, S. More

**Affiliations:** 1 Division of Nuclear Medicine, Department of Radiation Medicine, University of Cape Town, Cape Town, South Africa

## Abstract

**Background:**

Renal transplantation is a vital treatment for paediatric end-stage kidney disease (ESKD), but complications such as vascular thrombosis can threaten graft viability. Nuclear imaging plays a central role in evaluating graft function, with [^99m^Tc]Tc-MAG3 renogram commonly used and [^99m^Tc]Tc-DMSA scintigraphy applied less frequently. This case highlights the diagnostic complexity in a 7-year-old boy with ESKD secondary to posterior urethral valves (PUV), who underwent a deceased-donor renal transplant.

**Methods:**

Post-transplant imaging revealed conflicting results. On day 2, a MAG3 renogram showed no perfusion, uptake or excretion in the graft, suggesting vascular thrombosis. A repeat scan on day 6 confirmed absent function, with tracer activity attributed to the native kidney. Clinically, this was interpreted as severe delayed graft function. However, on day 14, a DMSA scan with single photon emission computed tomography (SPECT)/CT revealed cortical tracer uptake in the graft, indicating preserved parenchymal viability. This prompted a change in management, including continued monitoring and a planned renal biopsy.

**Results:**

The case underscores the complementary nature of MAG3 and DMSA imaging. MAG3 assesses tubular function and perfusion, while DMSA evaluates cortical integrity. Discrepancies between the two may reflect delayed perfusion recovery despite viable tissue, emphasising the importance of multimodal imaging in complex transplant scenarios.

**Conclusion:**

Ultimately, this case illustrates that early MAG3 findings of non-function do not always indicate irreversible graft failure. DMSA uptake can reveal viable tissue and potential for recovery, guiding more nuanced clinical decisions. Multimodal imaging, combined with clinical correlation, is essential in the early post-transplant period to avoid premature conclusions and support optimal patient outcomes.

# Abstract: Unveiling algorithm strengths: A deep dive into ordered subset expectation-maximisation and hyper dynamic penalised reconstruction algorithms

**Published:** 2026-07-28

**Authors:** P. Goorhoo, M. Qebetu, K.I. Banda, K. Legodi, C. Davis, L. Smith, A. Mdlophane, S. Sibiya, K. Mokoala, M. Sathekge, K. Ramonaheng

**Affiliations:** 1 Nuclear Medicine Research Infrastructure (NuMeRI), Pretoria, South Africa; 2 Department of Nuclear Medicine, University of Pretoria, Pretoria, South Africa

## Abstract

**Background:**

Accurate quantification in nuclear medicine is often limited by partial volume effects (PVEs), which cause underestimation of radiotracer uptake in small lesions. Digital positron emission tomography/computed tomography (PET/CT) systems – enhanced by LYSO crystals and silicon photomultipliers (SiPMs) – have improved spatial resolution and image quality, helping reduce PVEs. However, recovery coefficients (RCs) remain essential for partial volume correction (PVC). Advanced reconstruction algorithms such as 3D-ordered subset expectation-maximisation (3DOSEM), hyper iterative and hyper dynamic penalised reconstruction (DPR) further refine image quality. Hyper DPR, powered by a convolutional neural network, balances noise suppression, contrast and resolution. This study evaluates the quantitative performance of these reconstruction methods using RCs to assess accuracy and PVC suitability.

**Methods:**

PET/CT imaging was performed on the uMI 550 system, which integrates an 80-slice CT scanner with a high-resolution digital PET detector. A NEMA IEC body phantom with six spheres of varying diameters was filled with F-18 or Ga-68. Each scan lasted 15 min. VOIs were defined using fixed-threshold segmentation. Reconstructions were performed using ordered subset expectation maximisation (OSEM) (20 subsets, variable iterations) and hyper DPR with varying strengths. Quantitative analysis included SUVmean, RCs, image contrast, coefficient of variation (COV) and background variability (BV). A Jaszczak phantom was used to assess contrast and noise under identical parameters.

**Results:**

For F-18, the 37-mm sphere had an RC of 0.704 ± 0.0079 using OSEM (two iterations). The greatest RC difference between OSEM and hyper DPR was 7.69% ± 0.698 in the 10-mm sphere. Image contrast and SUV values decreased with smaller spheres. COV remained below 30% for 4–10 iterations but exceeded it at two iterations for smaller spheres. BV peaked at 10 iterations.

**Conclusion:**

Optimising reconstruction parameters improves PET/CT quantification. Hyper DPR enhances image quality, but balancing contrast recovery and noise is key for accurate diagnostics and effective PVC.

# Abstract: Bone scan as a cost-conscious screening strategy for high-risk prostate cancer: An audit from Klerksdorp/Tshepong Hospital Complex, South Africa

**Published:** 2026-07-28

**Authors:** L. Korowlay, I. Sealanyane, P. Makua, K. Sekhwela, D. Matontsi, H. Ulrich, A. Ayeni

**Affiliations:** 1 Nuclear Medicine Department, Klerksdorp/Tshepong Hospital Complex, Klerksdorp, South Africa; 2 Division of Nuclear Medicine, University of the Witwatersrand, Johannesburg, South Africa; 3 Division of Nuclear Medicine, University of Cape Town, Cape Town, South Africa

## Abstract

**Background:**

Prostate-specific Membrane Antigen (PSMA) positron emission tomography (PET) imaging has emerged as the gold standard for staging high-risk prostate cancer but remains relatively inaccessible in many South African public healthcare setting. Bone scintigraphy, because of its affordability and availability, is still widely used. This audit evaluated bone scans’ utility in identifying high-volume metastatic disease in high-risk prostate cancer, in cases where further PSMA-based imaging is unlikely to influence planned management decisions, to reduce diagnostic costs.

**Methods:**

A retrospective review of all bone scans (July 2023 – March 2025) for high-risk prostate cancer patients referred for staging/restaging at Klerksdorp/Tshepong Hospital Complex. Scans results were dichotomised into widespread metastases (including ‘superscans’), and others that include normal, equivocal, low-volume disease according to CHAARTED criteria.

**Results:**

A total of 732 bone scans were performed during the period under review: 400 (54.6%) for prostate cancer, including 138 high-risk cases. Among 138 high-risk patients (mean age 68, 52% Gleason ≥ 8, median PSA 100 ng/mL), bone scans identified widespread skeletal metastases in 36.2% (12.3% superscans). Thus, in our cohort, result of bone scintigraphy (in conjunction with diagnostic CT) allowed planned treatment to proceed without further PSMA imaging in over one third of patients with high-risk prostate cancer.

**Conclusion:**

In resource-constrained settings, a tiered diagnostic pathway starting with bone scan (plus CT) and reserving PSMA PET/CT for negative or ambiguous cases can offer a cost-effective and pragmatic solution, especially in hormone-sensitive metastatic prostate cancer.

However, PSMA-based imaging should be regarded as mandatory for potential metastasis-directed therapy (MDT) in presumed oligometastatic disease and in evaluation for theranostic eligibility in castration-resistant prostate cancer. Outcome-based validation is required to solidify this recommended streamlined diagnostic approach.

# Abstract: Development of robust radiolabelling and quality control analytical methods for diagnostic PET tracer ^68^GaPSMA-62

**Published:** 2026-07-28

**Authors:** T. Ratsakatika, M. Shungube, Y. Mzizi, R. Sebatana, C. Davis, S. Sibiya, M. Qebetu, K. Ramonaheng, M. Sathekge, S. Mdanda, A. Mdlophane

**Affiliations:** 1 Nuclear Medicine Research Infrastructure (NuMeRI), Pretoria, South Africa

## Abstract

**Background:**

Prostate-specific membrane antigen (PSMA) is a glycoprotein highly overexpressed in prostate cancer (PCa), making it a valuable target for diagnostic imaging, staging and metastasis detection. Radiopharmaceuticals labelled with Gallium-68 (^68^Ga) targeting PSMA must exhibit high radiochemical purity (RCP > 90%) and minimal impurities to ensure accurate PET imaging. This study focused on developing and optimising a radiolabelling procedure for ^68^Ga-PSMA-62, alongside robust analytical methods for quality control to ensure product stability and clinical suitability. This study aimed to establish a reliable radiolabelling protocol and analytical quality control methods for ^68^Ga-PSMA-62 for use in PET imaging of prostate cancer.

**Methods:**

The radiolabelling process involved dissolving the PSMA-62 precursor in 0.04M HCl, combining it with a sodium acetate/saline buffer (pH 7) and adding 1 mL of ^^68^GaCl_3_. The mixture was heated at 90°C for 10 min. Quality control included HPLC-UV for radiochemical identity and Radio-TLC for radiochemical purity. Additional assessments included radionuclidic purity, precursor and excipient quantification, sterility and endotoxin levels.

**Results:**

Reducing the heating time from 20 to 10 min improved product yield and minimised activity loss. Substituting HEPES with sodium acetate maintained comparable radiolabelling efficiency. C18 purification yielded ~ 95% of the radiochemical with ± 99% purity. Across 12 batches, RCP exceeded 98%, RCI surpassed 100% and free ^68^Ga remained below 5%. Imaging results demonstrated superior tumour uptake and PET/CT image quality compared to PSMA-11.

**Conclusion:**

The optimised radiolabelling and quality control methods for ^68^Ga-PSMA-62 produced a highly pure and stable radiopharmaceutical suitable for clinical PET imaging. The compound showed enhanced imaging performance, supporting its potential as a superior alternative for prostate cancer diagnostics.

# Abstract: Intraarterial hepatic SPECT/CT imaging using ^99m^Tc-MAA in preparation for radioembolisation with extrahepatic distribution

**Published:** 2026-07-28

**Authors:** N. Zondi-Mredlana, D. Pillay, Z.J. Zulu, A. Nxumalo, S.S. Mafuleka, N. Soni, G. Byadewell

**Affiliations:** 1 LakeSmit and partners, Department of Nuclear Medicine, Durban, South Africa; 2 Hopelands Cancer Centre, Durban, South Africa

## Abstract

**Background:**

Radioembolisation using ^90^Y-microspheres is a precision therapy for both primary and metastatic liver malignancies. A critical step in treatment planning involves nuclear imaging with ^99m^Tc-macroaggregated albumin (^99m^Tc- MAA) to assess hepatic arterial anatomy and detect pulmonary or gastrointestinal shunting. This pre-treatment evaluation helps to prevent serious complications such as radiation-induced pneumonitis or gastritis by identifying extrahepatic tracer distribution and guiding catheter placement.

**Methods:**

A 54-year-old man presented with abdominal pain and was diagnosed with colorectal cancer involving the sigmoid colon. Imaging revealed periportal lymphadenopathy and hepatic metastases. Following a sigmoid colectomy, he was referred to oncology in August 2023. Despite two lines of chemotherapy, his liver disease progressed to bilobar hepatic involvement. A multidisciplinary team recommended liver-directed therapy with radioembolisation.

Initial planning angiography under local anaesthesia showed a proper hepatic artery dividing into left and right branches. 185 MBq of ^99m^Tc-MAA was injected into the left hepatic artery. Imaging revealed not only adequate tracer uptake in segments 2 and 3 of the liver but also extrahepatic accumulation in the stomach wall because of the right gastric artery arising from the left hepatic artery. The hepatopulmonary shunt was initially measured at 17% and later corrected to 7% using third-party software.

**Results:**

To address the gastric uptake, repeat angiography was performed. A microcatheter was used to access the left hepatic artery and confirm the right gastric artery. Prophylactic coil embolisation was successfully completed. A second injection of 259 MBq ^99m^Tc-MAA showed no extrahepatic accumulation and confirmed safe tracer distribution. A split-lobar treatment plan was initiated, starting with the left lobe.

**Conclusion:**

This case demonstrates the essential role of nuclear medicine in radioembolisation planning. Scintigraphic and angiographic evaluation with ^99m^Tc-MAA ensures safe, targeted therapy by identifying extrahepatic risks and optimising catheter placement, ultimately improving treatment outcomes.

# Abstract: Evaluating the variance in percentage uptake functions of ^99m^Tc-pertechnetate thyroid studies using two different thyroid quantification methods

**Published:** 2026-07-28

**Authors:** B.N. Tshijolo, L. Lackay, M. Marais, T. Geldenhuys

**Affiliations:** 1 Department of Medical Imaging and Therapeutic Sciences, Cape Peninsula University of Technology, Cape Town, South Africa; 2 Groote Schuur Hospital, Cape Town, South Africa

## Abstract

**Background:**

Thyroid quantification using technetium-99m (^99m^Tc) pertechnetate is essential for diagnosing functional thyroid disorders such as hyperthyroidism and hypothyroidism. However, because of the lack of a globally standardised acquisition protocol, nuclear medicine departments use varying methods. Two widely recognised approaches are the No-syringe analysis method (NSAM) and the syringe analysis method (SAM). These differing protocols may yield variations in thyroid percentage uptake values. This study aimed to determine whether there is a significant variance in thyroid percentage uptake when using NSAM versus SAM. It also sought to identify whether a consistent ratio exists between the results produced by the two methods.

**Methods:**

A prospective, quantitative study was conducted at a tertiary academic hospital in the Western Cape. Eleven ^99m^Tc-pertechnetate thyroid scans were collected between August and September 2019 using convenience sampling. Both NSAM and SAM were performed consecutively for each patient without disrupting routine workflow. Data were processed using Hermes software to calculate thyroid percentage uptake. Statistical analysis included mean, standard deviation (s.d.), range, linear regression, Bland-Altman plots and a paired *t*-test.

**Results:**

The NSAM yielded a mean uptake of 7.78% with a range of 25.3%, while SAM produced a mean of 6.55% and a range of 22.1%. The NSAM showed higher variance (70.55%) and s.d. (8.4%) compared to SAM (52.48% variance and 7.24% s.d.). The Bland-Altman plot showed most values within acceptable agreement limits although four exceeded the upper threshold. The paired *t*-test produced a *t*-value of 2.64, exceeding the critical value of 2.228.

**Conclusion:**

Despite minor statistical differences, the study found no significant variance between NSAM and SAM. Both methods are reliable for calculating thyroid percentage uptake in ^99m^Tc-pertechnetate studies and can be used interchangeably in clinical practice.
